# Risk factors for severe COVID-19 infection and the impact of COVID-19 infection on disease progression among patients with AAV

**DOI:** 10.1007/s10238-024-01351-x

**Published:** 2024-04-29

**Authors:** Chen Wang, Zhi-Ying Li, Gui-Ping Jiang, Ming-Hui Zhao, Min Chen

**Affiliations:** 1 Renal Division, Department of Medicine, Peking University First Hospital, Peking University Institute of Nephrology, No.8 Xishiku Street, Xicheng District, Beijing, 100034 China; 2 Key Laboratory of Renal Disease, Ministry of Health of China, Beijing, China; 3 Key Laboratory of Chronic Kidney Disease Prevention and Treatment (Peking University), Ministry of Education, Beijing, China; 4 Renal Division, The People’s Hospital of Rongchang District, Chongqing, China; 5 Peking-Tsinghua Center for Life Sciences, Beijing, China

**Keywords:** COVID-19, ANCA, Vasculitis, Flare

## Abstract

To identify risk factors for COVID-19 infection and investigate the impact of COVID-19 infection on chronic kidney disease (CKD) progression and vasculitis flare in patients with antineutrophil cytoplasmic antibody (ANCA)-associated vasculitis (AAV). This cohort study retrospectively analyzed the prevalence and severity of COVID-19 infection in 276 patients with AAV who were followed up. Logistic regression was employed to estimate the risk of COVID-19 infection as well as CKD progression and vasculitis flare upon COVID-19 infection. During the 6-month observation period, 213 (77.2%) of 276 patients were diagnosed with COVID-19 infection. Of these 213 patients, 49 (23.0%) had a COVID-19-related inpatient admission, including 17 patients who died of COVID-19 infection. AAV patients with severe COVID-19 infection were more likely to be male (OR 1.921 [95% CI 1.020–3.619], *P* = 0.043), suffered from worse kidney function (serum creatinine [Scr], OR 1.901 [95% CI 1.345–2.687], *P* < 0.001), had higher C-reactive protein (CRP) (OR 1.054 [95% CI 1.010–1.101], *P* = 0.017) and less likely to have evidence of initial vaccination (OR 0.469 [95% CI 0.231–0.951], *P* = 0.036), and Scr and COVID-19 vaccination were proven to be significantly associated with severe COVID-19 infection even after multivariable adjustment. Severe COVID-19 infection was significantly associated with subsequent CKD progression (OR 7.929 [95% CI 2.030–30.961], *P* = 0.003) and vasculitis flare (OR 11.842 [95% CI 1.048–133.835], *P* = 0.046) among patients with AAV. AAV patients who were male, and with worse kidney function were more susceptible to severe COVID-19 infection, which subsequently increased the risk of CKD progression and vasculitis flare.

## Introduction

Antineutrophil cytoplasmic antibody (ANCA)-associated vasculitis (AAV) comprises granulomatosis with polyangiitis (GPA, previously known as Wegener’s granulomatosis), microscopic polyangiitis (MPA), and eosinophilic granulomatosis with polyangiitis (EGPA, previously known as Churg–Strauss syndrome) [1]. The kidney is one of the most common organs involved in AAV [2], and the outcomes in patients with AAV have improved significantly over the past decades, although a significant proportion of them still develop end-stage renal disease (ESRD) [3]. Another important issue of outcomes is relapse, which is associated with subsequent progression to ESRD [4].

Throughout the pandemic of severe acute respiratory syndrome by coronavirus-2 (SARS-CoV-2) disease (COVID-19), many studies have reported a greater risk of poor outcomes among patients with various immune-mediated inflammatory diseases [5]. Patients with preexisting autoimmune diseases may undergo disease flare after COVID-19 infection [6, 7]. Meanwhile, COVID-19 infection has been proposed as a trigger of several autoimmune diseases as well [8, 9].

There may be a reciprocal influence between AAV and COVID-19 infection. Patients with AAV have been reported to have a higher likelihood of developing severe COVID-19 [10]. Moreover, SARS-CoV-2 would even be an upstream trigger of AAV. For example, it was found by Vlachoyiannopoulos et al. that 13% of patients with critical COVID-19 pneumonia positivity of ANCA [11]; several studies reported cases developed after COVID-19 infection [12, 13]. However, the evidence about the impact of COVID-19 infection on the disease progression among patients with preexisting AAV is limited, in particular, disease flare and chronic kidney disease (CKD) progression. Thus, the purpose of this study was to identify risk factors for COVID-19 infection and to investigate the impact of COVID-19 infection on CKD progression and vasculitis flare in patients with AAV.

## Methods

### Study population

This study reviewed 276 Chinese patients with AAV, diagnosed at Peking University First Hospital from 1999 to 2022 and followed up regularly at the outpatient. All patients met the Chapel Hill Consensus Conference criteria for AAV [1]. Patients with secondary vasculitis or with other comorbid renal diseases were excluded. Treatment protocols were described previously [14]. This research was conducted in accordance with the Declaration of Helsinki and was approved by the Clinical Research Ethics Committee of the Peking University First Hospital. Written informed consent was obtained from each patient or the guardians.

### Diagnosis of COVID-19

The diagnosis of COVID-19 is based on the detection of SARS-CoV-2 using polymerase-chain-reaction (PCR) assay [15], as well as antibody, metagenomic testing, CT scan, laboratory assay or a presumptive diagnosis based on symptoms and epidemiological history by physicians. Patients meeting the above criteria were then assessed for a COVID-19 diagnosis during the study period (November 1, 2022–May 1, 2023).

The evaluation and management of COVID-19 depended on the severity of the disease, as patients with mild-to-moderate signs and symptoms typically recovered at home, while patients with severe COVID-19 are usually hospitalized for observation and supportive care [15]. Severe clinical manifestations included a respiratory rate higher than 30 breaths per minute, an oxygen saturation of less than 93%, while the patient was breathing ambient air, a ratio of partial pressure to fraction of inspiration oxygen (PaO_2_/FiO_2_) ≤ 300 mmHg and the lesion progression more than 50% within 24–48 h in the chest imaging. Once either condition is met, the case would be diagnosed as severe COVID-19 infection [16].

### Clinical data and biochemical parameters

All the clinical and laboratory data were, respectively, collected from the medical records of the patients, including age, gender, diagnosis, date of diagnosis, ANCA serotype, disease phenotype, organ involvement, immunosuppressive therapies, concomitant treatment, laboratory values (serum creatinine [Scr], C-reactive protein [CRP], erythrocyte sedimentation rate [ESR], white blood cell count [WBC], neutrophils, lymphocytes, hemoglobin, CD4+ T cells, CD8+ T cells, natural killer [NK] cells and serum albumin). The estimated glomerular filtration rate (eGFR) was calculated by the Chronic Kidney Disease Epidemiology Collaboration (CKD-EPI) equation [17]. The Birmingham vasculitis activity score system (BVAS) was used in evaluating the disease activity of AAV [18]. The clinical and biochemical parameters before infection were collected from these patients’ last visit before their first symptoms of COVID-19 infection, which ranged from one week to three months earlier.

### Outcomes

Two critical outcomes are CKD progression and AAV flare. CKD progression was defined as incident ESRD and/or decline in eGFR by ≥ 50%, as described previously [19]. AAV flare was defined as the re-occurrence of disease attributable to active vasculitis, or worsened disease activity [20]. The outcome events were recorded during a 6-month observation period commencing from the day of diagnosis of COVID-19 infection.

### Statistical analysis

Continuous variables with normal distribution were described by mean and standard deviation, while those with and non-normal distribution were described by median and interquartile range. Categorical variables were described by frequency and percentage. Univariate analysis was performed by the Kruskal–Wallis test, one-way analysis of variance (ANOVA), or the chi-square test, as appropriate. Correlation between quantitative variables was performed using Pearson’s (for data that were in normal distribution) or Spearman’s correlations (for data that were in skewed distribution). Logistic regression was performed to explore the associations between the assessed parameters. *P* < 0.05 was considered statistically significant. Statistical analysis was performed using SPSS 18.0 (Chicago, IL, USA).

## Results

### General data of the patients

A total of 276 patients with confirmed diagnosis of AAV were enrolled: 110 were male, and 166 were female, with a median age of 66.5 (range, 17–91) years at diagnosis. 213 (77.2%) of 276 patients had a diagnosis of COVID-19 infection. Of the 213 patients, 49 (23.0%) had severe COVID-19 infection, including 17 patients who died of COVID-19 infection. 133 (48.2%) of 276 patients received COVID-19 vaccinations, including 107 (38.8%) finished the initial vaccination by receiving two or more SARS-CoV-2 vaccines. The demographic and baseline data of the study population are summarized in Table 1.

**Table 1:** Demographic and baseline features of the study population

	AAV patients without COVID-19 infection	AAV patients with mild-to-moderate COVID-19 infection	AAV patients with severe COVID-19 infection	AAV patients dying of COVID-19 infection
Number	63	164	32	17
Age, y	67.7 ± 11.0	61.9 ± 13.4	63.6 ± 13.2	69.7 ± 10.4
Male/Female	21/42	64/100	11/21	14/3
Induction therapy/Maintenance therapy	8/55	12/152	6/26	0/17
Vaccination/Not vaccination	29/34	79/85	12/20	3/14
Dialysis/Not dialysis	6/57	23/141	6/26	3/14
Serum creatinine, mg/dl	1.78 ± 0.94	1.57 ± 0.74	2.14 ± 1.24	2.92 ± 1.67
eGFR, ml/min/1.73m^2^	43.0 ± 23.5	50.1 ± 26.5	37.0 ± 20.9	30.1 ± 21.3
MPO-ANCA/PR3-ANCA	57/7	142/24	25/5	14/2
ANCA titers, RU/ml	0 (0–104.8)	0 (0–79.0)	0 (0–103)	0 (0–56.0)
CRP, mg/l	1.0 (0.0–5.0)	2.0 (0.0–4.0)	1.0 (0.0–7.8)	4.5 (3.0–13.0)
WBC, *10^9^/l	8.2 ± 5.7	7.4 ± 2.7	7.1 ± 2.9	7.4 ± 1.3
Neutrophils, *10^9^/l	5.3 ± 3.6	4.8 ± 2.3	4.9 ± 2.1	5.0 ± 1.2
Lymphocytes, *10^9^/l	2.0 ± 1.1	1.8 ± 0.8	1.6 ± 1.3	1.5 ± 0.5
Monocytes, *10^9^/l	0.50 ± 0.19	0.61 ± 0.75	0.51 ± 0.20	0.47 ± 0.10
CD4+ T cells, counts/ml	793 ± 372	925 ± 530	658 ± 382	600 ± 256
Hemoglobin, g/dl	12.1 ± 1.6	12.5 ± 1.7	12.0 ± 2.1	11.7 ± 2.4
Serum albumin, g/l	39.7 ± 4.9	41.3 ± 3.2	39.3 ± 4.9	40.1 ± 4.2

*ANCA* antineutrophil cytoplasmic antibody, *CRP* C-reactive protein, *eGFR* estimated glomerular filtration rate, *WBC* white blood cell counts in peripheral blood

### Risk factors for severe COVID-19 infection in patients with AAV

For the risk factors of severe COVID-19 infection in patients with AAV, patients with severe COVID-19 infection were more likely to be male (OR 1.921 [95% CI 1.020–3.619], *P* = 0.043), suffer from worse kidney function (Scr, OR 1.901 [95% CI 1.345–2.687], *P* < 0.001; eGFR, OR 0.976 [95% CI 0.958–0.994], *P* = 0.009, respectively), and have higher CRP (OR 1.054 [95% CI 1.010–1.101], *P* = 0.017) and less likely to have initial vaccination (OR 0.469 [95% CI 0.231–0.951], *P* = 0.036), suggesting that vaccination had a significant protective effect among patients with AAV from severe COVID-19. Kidney function, and COVID-19 vaccination were significantly associated with severe COVID-19 infection in multivariable adjustment (Scr, adjusted OR 1.926 [95% CI 1.276–2.909], *P* = 0.002; initial COVID-19 vaccination, adjusted OR 0.212 [95% CI 0.046–0.969], *P* = 0.045, respectively).

For the risk factors of death from severe COVID-19 infection in patients with AAV, sex, Scr, CRP and COVID-19 vaccination were significant in unadjusted analysis, as patients with severe COVID-19 infection were more likely to be male (OR 11.958 [95% CI 2.661–53.746], *P* = 0.001) and have higher Scr (OR 2.311 [95% CI 1.313–4.065], *P* = 0.004) and CRP (OR 1.059 [95% CI 1.014–1.106], *P* = 0.009) and less likely to have vaccination (OR 4.773 [95% CI 1.063–21.437], *P* = 0.041). However, none of these factors was significantly associated with the mortality of severe COVID-19 infection in multivariable analysis.

### Impact of COVID-19 infection on CKD progression or vasculitis flare among patients with AAV

Nine patients suffered from CKD progression, and three patients got AAV flare upon COVID-19 infection within three weeks. The clinical and biochemical parameters before and after COVID-19 infection are summarized in Table 2, including kidney function, disease activity of AAV, inflammatory indicators, various blood cells counts and nutrition condition.

**Table 2:** Clinical and biochemical parameters before and after COVID-19 infection

	AAV patients without COVID-19 infection (n = 42)			AAV patients with mild-to-moderate COVID-19 infection (n = 111)			AAV patients with severe COVID-19 infection (n = 26)		
	Baseline	Follow-up	*P* values	Baseline	Follow-up	*P* values	Baseline	Follow-up	*P* values
Serum creatinine, mg/dl	1.62 ± 0.79	1.66 ± 1.04	0.545	1.56 ± 0.74	1.63 ± 0.82	**0.038**	1.92 ± 0.99	2.16 ± 1.36	**0.020**
eGFR, ml/min/1.73m^2^	46.1 ± 23.6	47.0 ± 24.6	0.454	50.9 ± 27.6	49.0 ± 26.2	0.094	39.1 ± 20.3	37.9 ± 14.6	0.050
Urine RBC, counts/HP	1.4(0.4–5.1)	1.0(0.0–1.0)	0.195	1.0(0.0–2.0)	0.9(0.4–2.9)	0.256	1.1(0.4–8.3)	0.8(0.3–3.3)	0.528
ANCA titers, RU/ml	20(0–102)	32(0–143)	0.209	0(0–80)	21(0–88)	0.574	30(0–100)	0(0–105)	0.123
CRP, mg/l	1.0(0.0–5.0)	2.0(0.0–6.3)	0.847	2.0(0.0–4.0)	2.0(0.0–4.6)	0.285	1.0(0.0–7.8)	5.0(0.0–14.5)	**0.021**
ESR, mm/h	27.0 ± 15.9	30.3 ± 22.5	0.193	26.7 ± 21.7	33.9 ± 26.9	**0.001**	22.4 ± 14.7	43.0 ± 29.0	**0.005**
WBC, *10^9^/l	8.1 ± 6.2	7.3 ± 2.3	0.396	7.3 ± 2.6	7.4 ± 2.7	0.305	6.6 ± 1.8	7.8 ± 3.0	**0.017**
Neutrophils, *10^9^/l	5.2 ± 3.7	4.8 ± 2.0	0.416	4.8 ± 2.2	5.0 ± 2.3	0.230	4.6 ± 1.8	5.5 ± 2.6	**0.008**
Lymphocytes, *10^9^/l	2.0 ± 1.2	1.9 ± 1.0	0.780	1.8 ± 0.7	1.8 ± 0.8	0.830	1.5 ± 0.9	15 ± 0.6	0.883
Monocytes, *10^9^/l	0.47 ± 0.17	0.52 ± 0.20	0.045	0.55 ± 0.40	0.55 ± 0.34	0.707	0.48 ± 0.16	0.62 ± 0.21	**0.002**
Platelets, *10^9^/l	213 ± 60	218 ± 54	0.320	215 ± 49	227 ± 67	**0.012**	192 ± 59	192 ± 64	0.993
CD4+ T cells, counts/ml	470 ± 349	580 ± 187	0.352	851 ± 340	795 ± 324	0.130	553 ± 149	657 ± 211	0.259
CD8+ T cells, counts/ml	690 ± 417	958 ± 569	0.093	898 ± 334	918 ± 439	0.699	623 ± 268	752 ± 358	0.091
NK cells, counts/ml	141 ± 45	280 ± 156	0.182	513 ± 384	547 ± 351	0.527	211 ± 21	333 ± 212	0.124
Hemoglobin, g/l	123.4 ± 13.8	122.7 ± 17.2	0.733	125.4 ± 16.0	124.8 ± 30.0	0.809	123.4 ± 13.8	122.7 ± 17.2	0.756
Serum albumin, g/l	41.0 ± 3.6	40.1 ± 3.8	**0.011**	41.4 ± 3.3	40.8 ± 3.6	**0.010**	41.0 ± 3.6	40.1 ± 3.8	**0.031**

*ESR* erythrocyte sedimentation rate, *NK* natural killer, *RBC* red blood cell.   *P*<0.05 are considered statistically significant and highlighted in bold.

The impact of COVID-19 infection on the risk of CKD progression or vasculitis flare among patients with AAV was analyzed. Mild-to-moderate COVID-19 infection did not show significant impact on CKD progression or vasculitis flare among patients with AAV, while severe COVID-19 infection had a significant impact on the risk of CKD progression (OR 7.929 [95% CI 2.030–30.961], *P* = 0.003) and vasculitis flare (OR 11.842 [95% CI 1.048–133.835], *P* = 0.046) among patients with AAV. The clinical characteristics and outcomes of the three patients who suffered from AAV flare after COVID-19 infection are summarized in Table 3.

**Table 3:** Clinical characteristics and outcomes of patients suffering from AAV flare after COVID-19 infection

	Age/Sex	Severe COVID-19	Vaccination status	Time between COVID-19 infection and AAV flare	Symptoms	Serum creatinine, mg/dl	ANCA type/titers	BVAS	Therapy	Outcome
Patient 1	51/F	Yes	three shots	14 days	Fever, fatigue, cough and hemoptysis	1.21	PR3-ANCA 105 RU/ml	18	Pulse methylprednisolone, prednisolone, CTX	AAV remission
Patient 2	44/F	Yes	three shots	15 days	Fever, cough and dyspnea	4.55	negative	20	plasmapheresis, pulse methylprednisolone, prednisolone, RTX	AAV remission, ESRD
Patient 3	75/F	Yes	two shots	20 days	Fever, cough and throat pain	1.35	MPO-ANCA > 200 RU/ml	12	Pulse methylprednisolone, prednisolone, CTX	AAV remission, CKD progression

*BVAS* Birmingham vasculitis activity score, *CKD* chronic kidney disease, *CTX* cyclophosphamide, *ESRD* end-stage renal disease, *RTX* rituximab

Of the nine patients who suffered from CKD progression, six finished the initial vaccination by getting two or three shots of SARS-CoV-2 vaccines, while the other three were unvaccinated. Neither getting COVID-19 vaccination nor finishing the initial vaccination shows a significant effect on CKD progression (OR 2.906 [95% CI 0.735–11.493], *P* = 0.128; OR 3.640 [95% CI 0.920–14.405], *P* = 0.066, respectively). All three patients who got AAV flare upon COVID-19 infection finished the initial vaccination (Table 3), neither getting COVID-19 vaccination nor finishing the initial vaccination showed a significant effect on AAV flare.

## Discussion

This current study is a large-scale real-world study to identify risk factors for severe COVID-19 infection and investigate the impact of COVID-19 infection on CKD progression and vasculitis flare among patients with AAV. Our results showed in AAV patients, those with severe COVID-19 infection required hospitalization, and those who died of COVID-19 infection were more likely to be male, with poor kidney function, and were less likely to have the vaccination. Meanwhile, severe COVID-19 infection significantly increased the risk of CKD progression and vasculitis flare in AAV patients.

The COVID-19 pandemic poses challenges in the management of diseases including both CKD and AAV. According to our results, there seemed to be a vicious circle between kidney dysfunction and severe COVID-19 infection. Consistently, Jdiaa et al. demonstrated an increased risk of mortality and hospitalization in patients with preexisting CKD when they had COVID-19 infection [21]. Therefore, prophylactic measures and critical care management would be of importance to patients with CKD who are susceptible to COVID-19 infection.

As above mentioned, flares of preexisting autoimmune diseases in patients with COVID-19 have been reported, including systemic lupus erythematosus (SLE) and IgA vasculitis [6, 7]. In the current study, severe COVID-19 infection increased the risk of vasculitis flare among patients with AAV. The underlying mechanism might involve autoimmune conditions activation triggered by a hyper-inflammatory state and viral persistence in SARS-CoV-2 infection [22]. First, elevated neutrophil extracellular nets (NETs) formation and excessive NETosis, referring to the process of neutrophil death and NETs releasing, have been observed and drawn great attention in COVID-19 infection [23–25]. Second, complement activation in COVID-19 has been shown to interact with platelet activation and NETosis [26–28]. Last but not least, the virus–host interactions may lead to both direct and indirect microvasculature damage through endothelial cell inflammation [29], thus playing as a ‘trigger factor’ for the onset or flare-up of AAV, all the above mechanisms also play essential roles in the pathogenesis of AAV [30], and more data are needed to establish solid evidence about this subject.

Vaccines are considered the ‘game changer’ of the pandemic and are effective in the general population in preventing transmission, severe disease courses and mortality due to COVID-19 [31]. The current study indicated that vaccination had a significant protective effect on AAV patients from severe COVID-19 infection or dying of COVID-19 infection. What needs to be illustrated is that the protective effect would be significantly exerted only by receiving two or more shots of SARS-CoV-2 vaccines to finish the initial vaccination. As two sides of the same coin, it is worth noting that several newly onset or relapsing AAV cases following COVID-19 vaccination have also been reported [32–35]. In our cohort, one patient with newly onset AAV after COVID-19 vaccination was included, presenting with severe disease including acute kidney injury. Thus, more data are needed to weigh the risk of a relapse against the benefits of COVID-19 vaccination.

The limitation of the current study was the need for more data about the general population and control group of non-AAV COVID-19 patients. According to published reports about the general population, there were several shared risk factors for severe COVID-19 infection, such as aging, male gender, renal failure, specific comorbidities, immunocompromised status, and being unvaccinated [36, 37]. According to another study on patients with SLE, the main risk factors for severe COVID-19 infection included receiving rituximab treatment, renal failure, complicated hypertension and being unvaccinated [38]. Thus, these shared risk factors among different populations underscore the importance of age, gender, renal function, and vaccination status in predicting the severity of COVID-19 infection.

## Conclusions

AAV patients who were male with worse kidney function and without COVID-19 vaccination were more susceptible to severe COVID-19 infection, which in turn increased the risk of CKD progression and vasculitis flare among patients with AAV.

## Data Availability

The data that support the findings of this study are available from the corresponding author on request.

## Abbreviations

AAV: ANCA-associated vasculitis
ANCA: Antineutrophil cytoplasmic antibody
ANOVA: One-way analysis of variance
BVAS: Birmingham vasculitis activity score system
CKD: Chronic kidney disease
CKD-EPI: Chronic Kidney Disease Epidemiology Collaboration
CRP: C-reactive protein
eGFR: Estimated glomerular filtration rate
EGPA: Eosinophilic granulomatosis with polyangiitis
ESRD: End-stage renal disease
ESR: Erythrocyte sedimentation rate
GPA: Granulomatosis with polyangiitis
MPA: Microscopic polyangiitis
NK: Natural killer
PaO_2_/FiO_2_: Ratio of partial pressure to fraction of inspiration oxygen
PCR: Polymerase-chain-reaction
SARS-CoV-2: Severe acute respiratory syndrome by coronavirus-2
Scr: Serum creatinine
SLE: Systemic lupus erythematosus WBC, white blood cell count
